# Income Raising for Voluntary Hospitals

**Published:** 1913-07-26

**Authors:** 


### The Hospital

The Workers' Newspaper of Administrative Medicine and Institutional Life, Administration, National Insurance and Health.

No. 1413, Vol. LIV. SATURDAY, JULY 26, 1913. PRICE ONE PENNY.

### INCOME RAISING FOR VOLUNTARY HOSPITALS.

In our columns, and notably in the columns of *Truth*, protest has properly been made against the expenditure which was incurred last year by the promoters of Alexandra Day during the first year, when something like 30 per cent. of the £18,000 brought to credit was expended on the cost of collection and kindred charges. This result has been criticised justly as unsatisfactory, and a few weeks ago the *Saturday Review* published a somewhat scathing but useful article in relation to this matter. This article has elicited a correspondence which reveals certain weaknesses in connection with voluntary effort for the hospitals, and also exhibits the sources of strength which the audited accounts of some voluntary hospitals show under this head year by year. Mr. Sydney Holland took up the challenge of the *Saturday Review*, which invited hospital managers to express their opinion on the Alexandra Day collections. He entered into a warm defence of the methods pursued, and in his enthusiasm was led to make one or two statements which may harmfully affect the credit of the financial methods of the voluntary hospitals with the giving public. He illustrates the wrong methods of attempting to raise money by voluntary hospitals by recording that a whole-page advertisement in all the leading papers in London, which probably entailed an expenditure of more than £1,000, only brought in £900. He inferred that in his view an expenditure of 50 per cent. on the total sum received was good business by stating that in private life, when an investment of £100 can obtain at once a return of £200, he would think he had not done badly. He asks, further, "Do you know that it takes one hundred miles of writing, many more of typing, to get £50?" The editor of the *Saturday Review* and some of his correspondents have criticised these statements, and we venture to express the hope that Mr. Holland on consideration may take the opportunity in the course of his reply to modify them, because, as they stand, they are calculated to create a wrong impression in the public mind as to the actual cost of raising money for the voluntary hospitals.

The *Saturday Review* insists that Mr. Holland's theory of hospital finance is natural and simple. "So long as he gets the money for his hospital, it does not concern him what it costs to get it or why people give it. If the expenses on Rose Day eat up 90 per cent. of the takings, Mr. Holland would be grateful for the balance. That may be quite right from Mr. Holland's point of view, but it is very bad finance and very false economy for the country. If Mr. Holland is correct, which we cannot doubt, the whole odious and wasteful business of voluntary money raising ought to be swept away. If there is anything which it is the duty of the whole nation to support it is hospitals. The energy and self-devotion which is now thriftlessly expended on begging could then be diverted to better uses." The foregoing extract proves the necessity and desirability for Mr. Holland, in view of the average ascertained cost of raising money for the voluntary hospitals, to adopt our suggestion. Mr. Michelli, of the "Dreadnought" Hospital, who has proved himself to be a most successful raiser of revenue for voluntary hospitals, declares "as a matter of fact the proportion of cost of appeal work to the total income of hospitals is nearer 5 per cent. than the 90 per cent. mentioned by the *Saturday Review*." He points out, further, that he estimates "one hundred miles of writing means about 60,000 letters, and if Mr. Holland really gets only £50 in reply he must be singularly unfortunate." The postage on 60,000 letters would amount to £250, and no institution, whether voluntary or otherwise, and no business enterprise, could exist which kept on spending £250 to earn £50.

The secret of successful appeal in the present day consists in finding constituents who are known to take, or ought to take, a personal interest in the work of the particular hospital which asks them for a contribution. That is the policy which has made Mr. Michelli so eminently successful, and where appeals are scattered more widely and more indiscriminately the returns must become so small as to be ruinous in the end. Mr. Holland does less than justice to the results produced at the London Hospital by its appeals to the public for funds. Thus in the year 1912 the expenditure at the London, Mr.

Michelli states, was only £1,709, against an income, exclusive of investments, of £82,000. All honour to Mr. Holland for his zeal and enthusiasm on behalf of the London Hospital and the amount of time and energy he puts into the work. It is hard work like his which encourages other workers for the voluntary system, but in justice to that work it is of the first importance that every statement made in connection with it should be capable of proof on an actuarial basis. Otherwise misapprehension is sure to be caused, prejudice is excited, and the difficult task of raising money must be made unnecessarily harder and harder every year.

The greater the zeal and the more widespread the efforts of an individual man on behalf of the hospitals the more essential is it that he should recognise the importance of self-discipline to secure that no utterance or act of his shall ever do anything to increase the difficulties of his fellow-workers in the hospital field, upon whom may devolve the responsibility of obtaining the necessary money from the public. In fact, it is not the case that any voluntary hospital could continue to live in the present day if its managers spent on advertisements and appeals 50 or even 25 per cent. of the voluntary revenue paid to it by the public. The necessary funds can and ought to be raised for our voluntary hospitals at a cost of between 10 and 15 per cent., with a maximum in extreme cases of 15 per cent. No institution should be dependent mainly upon the exertions of one individual for its voluntary revenue, for such a practice in the end means disaster. We know from our own personal experience the truth of this principle, and we have made it a rule to enforce it in raising money for hospitals, a business which requires training, knowledge, and special gifts. Given these, the raising of money for this purpose, in England at any rate, can be made to prove in practice the most economical of financial transactions in the business sense.

